# Combined laparoscopic ovariectomy and laparoscopic‐assisted gastropexy versus combined laparoscopic ovariectomy and total laparoscopic gastropexy: A comparison of surgical time, complications and postoperative pain in dogs

**DOI:** 10.1002/vms3.249

**Published:** 2020-02-03

**Authors:** Fabio Leonardi, Roberto Properzi, Jessica Rosa, Paolo Boschi, Silvia Paviolo, Giovanna L. Costa, Cristiano Bendinelli

**Affiliations:** ^1^ Department of Veterinary Science University of Parma Parma Italy; ^2^ Private Practitioner Rapallo Italy; ^3^ Department of Veterinary Science University of Messina Polo Universitario dell’Annunziata Messina Italy

**Keywords:** analgesia, dog, laparoscopic gastropexy, laparoscopic ovariectomy, postoperative pain

## Abstract

The trend in laparoscopy is to develop easy and rapid techniques associated with reduced intraoperative complications and decreased postoperative pain. The aim of this study was to compare combined laparoscopic ovariectomy (OIE) and laparoscopic‐assisted incisional gastropexy (LAG) with combined laparoscopic OIE and total laparoscopic gastropexy (TLG) for surgical time, incidence of complications and postoperative pain. Twenty‐eight female dogs were randomly assigned to the LAG group (*n* = 14) or the TLG group (*n* = 14). All laparoscopic procedures were performed using a three‐port technique. The gastropexy was located 3 cm caudal to the 13th rib and 4 cm lateral to the rectus abdominis muscle. Surgical time (minutes [min]), intraoperative complications and postoperative complications were recorded. The Glasgow pain score (GPS) (short form) was calculated before surgery and at 1, 6, 12, 18 and 24 hr after extubation. Surgical time was significantly longer in the TLG group (48 ± 2 min) compared with the LAG group (39 ± 2 min). Minor postoperative complications occurred in both groups and included swelling (*n* = 2) and subcutaneous emphysema (*n* = 1). No significant differences regarding the GPS were recorded between groups. The GPS was significantly higher in both groups at 1 hr and 6 hr than before surgery. Two dogs in each group required rescue analgesia. Combined laparoscopic OIE and TLG require more time to perform than combined laparoscopic OIE and LAG. Neither procedure results in significant surgical complications. Postoperative pain for 24 hr was mild and comparable in both groups.

## Graphical abstract

Combined laparoscopic ovariectomy (OIE) and total laparoscopic gastropexy (TLG) requires more time to perform than combined laparoscopic OIE and laparo‐assisted gastropexy (LAG). Neither procedure results in significant surgical complications. Postoperative pain for 24 hr was mild and comparable in dogs undergoing combined OIE and TLG or combined OIE and LAG.

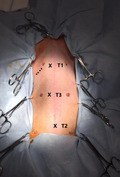

## 1 INTRODUCTION

Prophylactic gastropexy is the standard of care for the prevention of gastric dilatation and volvulus in dogs (Glickman, Lantz, Schellenberg, & Glickman, 1998; Rasmussen, 2003). Gastropexy creates a permanent adhesion between the stomach antrum and the right body wall preventing volvulus (Rasmussen, 2003). Minimally invasive gastropexy techniques reduce postoperative pain and inflammation of the incision site, ensure a rapid return to normal activity and are associated with high client satisfaction (Haraguchi et al., 2017; Loy Son et al., 2016; Mayhew & Cimino, 2009; Rasmussen, 2003). Minimally invasive gastropexy techniques can be successfully combined with elective ovariectomy (OIE), which is a procedure commonly performed in veterinary practice (Bendinelli, Properzi, et al., 2019; Gandini & Giusto, 2016; Rivier, Furneaux, & Viguier, 2011; Runge & Mayhew, 2013).

Minimally invasive gastropexy techniques include laparoscopic‐assisted gastropexy (LAG) techniques, total laparoscopic gastropexy (TLG) techniques and endoscopically assisted techniques (Dujowich, Keller, & Reimer, 2010; Mathon et al., 2009; Rasmussen, 2003; Rivier et al., 2011; Takacs et al., 2017). Laparoscopic single‐port or multi‐port techniques are available for performing combined ovariectomy and LAG or TLG in dogs (Bendinelli, Properzi, et al., 2019; Gandini & Giusto, 2016; Rivier et al., 2011; Runge & Mayhew, 2013). LAG techniques require exteriorization of the stomach through an incision in the body wall (Haraguchi et al., 2017) or only skin incision and subcutis dissection along the line of the peritoneal electrocoagulation (Mathon et al., 2009), and gastropexy is performed using extra‐abdominal suturing (Haraguchi et al., 2017; Mathon et al., 2009; Rivier et al., 2011). With TLG techniques, gastropexy is carried out completely laparoscopically using endoscopic suture‐assist devices, linear endoscopic stapling devices and intracorporeal suturing with knot tying or knotless barbed sutures (Coleman & Monnet, 2017; Deroy et al., 2019; Hardie et al., 1996; Mayhew & Cimino, 2009; Spah et al., 2013; Takacs et al., 2017). TLG requires practice to perform efficiently, and it is commonly associated with extended surgical times compared with LAG (Mayhew & Cimino, 2009).

Various intraoperative complications (e.g. organ laceration, haemorrhage, difficulty in removing ovarian tissue and spontaneous pneumothorax) may occur with both laparoscopic gastropexy and laparoscopic OIE (Bendinelli, Leonardi, & Properzi, 2019; Coleman & Monnet, 2017; Hardie et al., 1996; Mathon et al., 2009; Mayhew & Cimino, 2009; Properzi, Boschi, & Leonardi, 2018; Runge & Mayhew, 2013; Spah et al., 2013), whereas additional intraoperative technical difficulties are described with regard to TLG (e.g. breakage of the suture, inadequate length of the suture and suture knotted prematurely) (Mayhew & Cimino, 2009; Spah et al., 2013; Takacs et al., 2017). Nevertheless, TLG is superior to LAG in terms of reduced postoperative pain and decreased incidence of postoperative complications (Mayhew & Cimino, 2009).

Perioperative pain management is highly important when treating animals undergoing surgery (Bendinelli, Properzi, et al., 2019; Costa, Nastasi, Spadola, Leonardi, & Interlandi, 2019; Haraguchi et al., 2017). Although LAG, TLG and laparoscopic OIE are minimally invasive procedures, postoperative pain mainly occurs over the first 24 hr after surgery (Bendinelli, Properzi, et al., 2019; Haraguchi et al., 2017; Mayhew & Cimino, 2009; Rivier et al., 2011). Prevention, evaluation and treatment of postoperative pain are critical because uncontrolled pain might lead to cardiovascular stress, immunosuppression and anorexia (Hancock et al., 2005).

The aim of this study was to compare surgical time, incidence of intraoperative and postoperative complications and short‐term postoperative pain in dogs for combined laparoscopic OIE and LAG versus combined laparoscopic OIE and TLG. Based on the veterinary literature, we hypothesized that combined laparoscopic OIE and TLG was associated with extended surgical time, decreased incidence of complications and reduced postoperative pain compared with combined laparoscopic OIE and LAG.

## 2 MATERIALS AND METHODS

### 2.1 Animals

This study was performed in accordance with the animal welfare legislation and was approved by the Institutional Ethics Committee for animal welfare. All owners were informed in detail about the study design and signed the consent form.

Animals enrolled in the study were sexually intact female large‐breed dogs.

The inclusion criteria were as follows: dogs undergoing combined laparoscopic OIE and laparoscopic gastropexy, age ≥ 6 months, body condition score of 3/5, and healthy dogs that demonstrated no abnormalities on physical examination (cardiac and thoracic auscultation, heart and respiratory rates, systolic blood pressure and body temperature) and had normal complete blood count (erythrocytes, leukocytes, haemoglobin, haematocrit and platelets) and normal biochemical parameters (urea, creatinine, glucose, total bilirubin, gamma‐glutamyl transferase, total proteins and albumin).

The exclusion criteria were as follows: previous abdominal surgery and pharmacological treatments, pregnant or lactating in the previous 60 days (d).

The patients were randomly assigned until 14 dogs were in each group (combined laparoscopic OIE and laparoscopic‐assisted gastropexy, LAG group, or combined laparoscopic OIE and total laparoscopic gastropexy, TLG group) using SAS software version 9.4 (SAS Institute Inc., Cary).

It was determined that at least 10 dogs needed to be included in each group to have an 80% power of detecting differences (*p* < .05) in surgical time, incidence of complications and pain score between groups.

### 2.2 Anaesthetic protocol

A standardized preoperative anaesthetic management was used. Food and water were withheld for 12 hr. Dogs were administered a combination of dexmedetomidine (Dexdomitor, OrionPharma) 4 µg/kg and methadone (Semfortan, Dechra) 0.2 mg/kg intramuscularly. Approximately 20 min after premedication, an intravenous catheter was placed in the right cephalic vein. General anaesthesia was induced with 2 mg/kg of propofol (Proposure, Merial) intravenously (IV). Then, endotracheal intubation was performed, and general anaesthesia was maintained with isoflurane (Isoflo, Ecuphar) delivered in 100% oxygen via a rebreathing circuit. Intraoperative analgesia was achieved by an IV loading dose of 0.2 µg/kg of sufentanil (Hameln Pharma Plus GmbH) followed by a 0.5 µg kg^‐1^ hr^‐1^ rate infusion kept constant until the end of surgery. Intraoperative monitoring was performed using a multiparametric monitor (Infinity Gamma XL, Scio four Oxi plus, Dräger). Mechanical ventilation supported respiration during surgery by intermittent positive pressure ventilation to maintain EtCO_2_ in a range 30−45 mmHg. The mechanical ventilator was set using the following parameters: tidal volume of 10–12 ml/kg, respiratory rates of 10–12 breaths per minute and ventilation pressure of 15–18 mmHg. Lactated ringer's solution (Acme) was administered by a 10 ml kg^−1^ hr^‐1^ constant rate infusion during surgery. All dogs received cefazolin (Teva) 22 mg/kg IV and meloxicam (Metacam, Boehringer Ingelheim) 0.2 mg/kg subcutaneously immediately before induction of anaesthesia.

### 2.3 Surgical procedures

**Figure 1:**
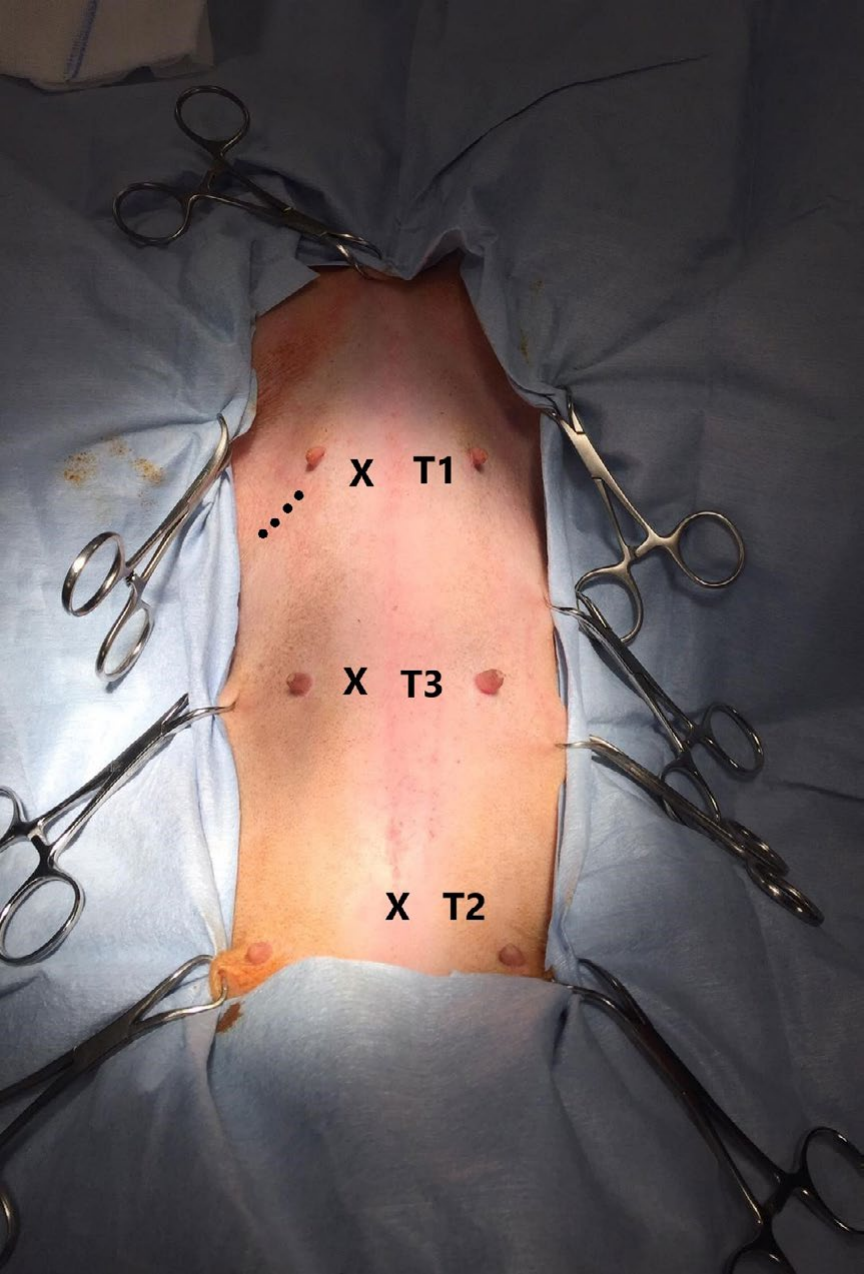
Port location (T1, T2 and T3) indicated by the symbol “X”. T1 was inserted 2–3 cm caudal to the xiphoid process of the sternum in the right paramedian position. T2 was inserted 2–3 cm caudal to the umbilicus on midline. T3 was inserted between T1 and T2 in the right paramedian position. Dotted line indicates the location of the skin incision and subcutis dissection

The same surgeon with 15 years of clinical experience in laparoscopy performed all laparoscopic procedures using a three‐port technique comparable to previously reported techniques (Mathon et al., 2009). All dogs were positioned in dorsal recumbency. Using the open (Hasson) technique, a 10‐mm cannula (T1) (Trocar X‐ONE, MedLine) was inserted 2–3 cm caudal to the xiphoid process of the sternum in the right paramedian position to create pneumoperitoneum using carbon dioxide by an automatic insufflator (264305 20, Karl Storz Endoscopy) with a pressure of 10–12 mmHg and to insert a 30° telescope measuring 10 mm in diameter and 31 cm in length (Hopkins II, Karl Storz Endoscopy). A 12‐mm cannula (T2) (Trocar X‐ONE) was inserted 2–3 cm caudal to the umbilicus on midline under endoscopic guidance. Then, a 5‐mm cannula (T3) (Trocar X‐ONE) was inserted between T1 and T2 in the right paramedian position (Figure 1).

**Figure 2:**
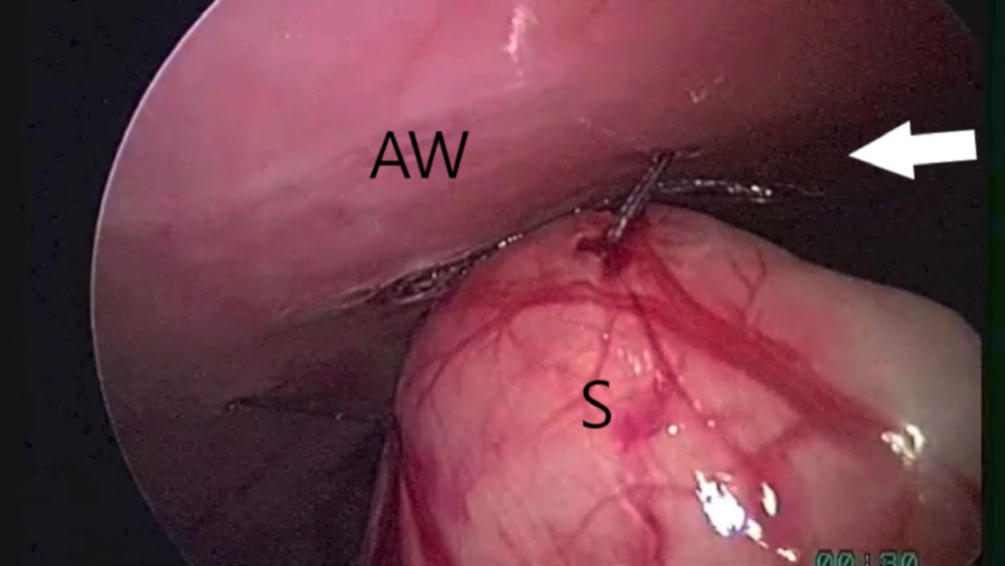
Laparoscopic‐assisted gastropexy. Stomach sutured to the abdominal wall with extracorporeal continuous sutures (arrow). The letters S and AW indicate the stomach and the abdominal wall respectively

At first, gastropexy was performed. In the LAG group, an approximately 3 cm gastropexy was performed with an extracorporeal suture (Mathon et al., 2009). Wolf grasping forceps (EndoorC, 611‐005‐00, Tontarra) were introduced in T2 to grasp the stomach between the greater and lesser curvatures at a distance of 4–5 cm from the pyloric antrum. The stomach was lifted towards the right, ventral abdominal wall. The pyloric antrum was shifted approximately 4 cm to the right costal arch. Five aligned electrocoagulation spots of approximately 0.3–0.5 mm (0.5 s long, 8 W power) were made on the peritoneal surface located 3 cm caudal to the 13th rib and 4 cm lateral to the right rectus abdominis muscle using an electrosurgical monopolar hook scalpel (Alsatom SU 140, Alsa) introduced through T3. The distance between each spot was approximately 0.5–0.8 cm. An approximately 5‐cm skin incision (Figure 1) was made along the line of the peritoneal electrocoagulation spots while transilluminating the area from inside the abdomen. After the skin incision and subcutis dissection, the stomach was sutured to the abdominal wall along the incision line with four extracorporeal sutures of 0 polyglactin 910 (Vicryl, Ethicon) in a continuous fashion (Rivier et al., 2011) (Figure 2). The needle was passed through the abdominal wall, next to the most cranial electrocoagulation spot, through the seromuscular layer of the antrum and back through the abdominal wall and skin incision (Bendinelli, Properzi, et al., 2019; Mathon et al., 2009).

**Figure 3:**
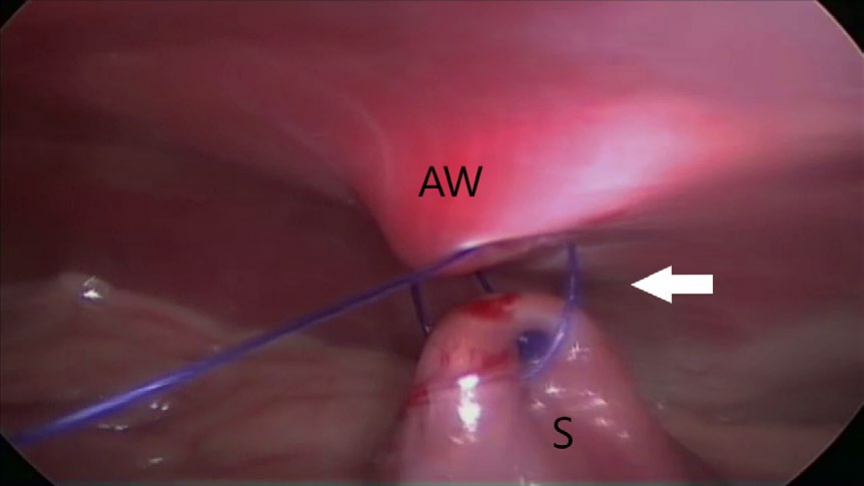
Total laparoscopic gastropexy. Stomach was anchored with the first two stitches (arrow) at the abdominal wall. The letters S and AW indicate the stomach and the abdominal wall respectively

**Figure 4:**
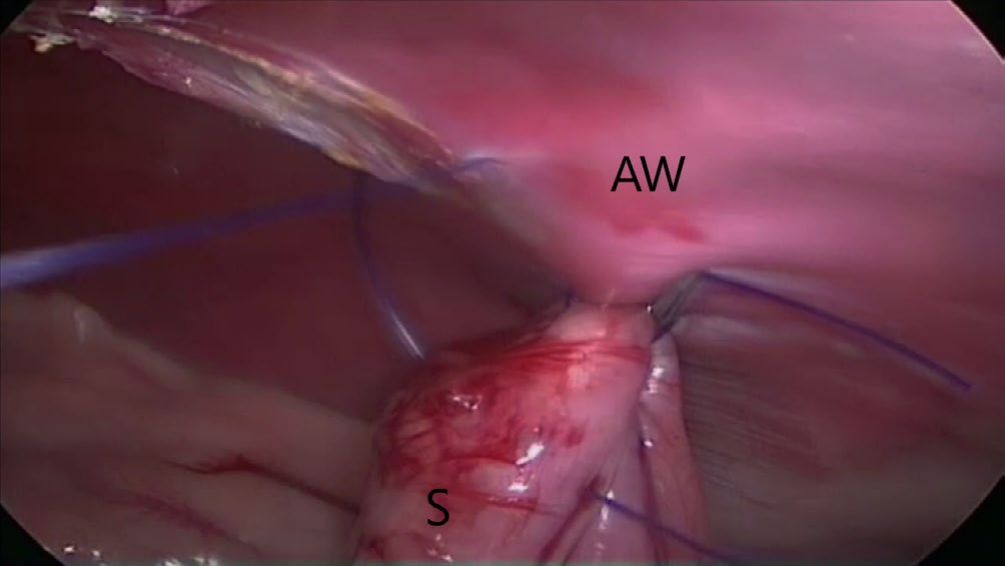
Total laparoscopic gastropexy. Suture passes through the antrum and the abdominal wall, next to the incision line. The letters S and AW indicate the stomach and the abdominal wall respectively

In the TLG group, an approximately 3‐cm gastropexy was performed via intracorporeal suturing with unidirectional barbed suture (Deroy et al., 2019). The self‐locking 0 polydioxanone monofilament barbed suture (HSRG 36 mm‐1/2 tapper cutting, Assut Europe) was inserted into the abdomen with a laparoscopic needle holder (614‐120‐53 Stainless 581, Tontarra) through T3. A second needle holder (614–120–53 Stainless 581, Tontarra) was inserted into the abdomen through T2. At first, the stomach was anchored with the first two bites at the abdominal wall in a right paracostal position 3 cm caudal to the 13th rib (Figure 3). An approximately 3‐cm incision was made on the peritoneal surface located 3 cm caudal to the 13th rib and 4 cm lateral to the right rectus abdominis muscle using an electrosurgical monopolar hook scalpel (Alsatom SU 140, Alsa) introduced through T3. Then, the suture was started between the greater and lesser curvatures of the stomach without incising the seromuscular layer of the stomach (Deroy et al., 2019). The barbed suture passed through the seromuscular layer of the antrum and through the abdominal wall, entering and exiting next to the incision line (Figure 4). Subsequent bites were taken 3–5 mm apart. After each bite, the suture was pulled taut to anchor the barbs in the tissue, and the simple continuous suture was completed (Takacs et al., 2017). The suture was reinforced with an endoclip (Ligamax 5 Med/Large, Ethicon) (Palmisano et al., 2014). The redundant end of the barbed suture was cut using laparoscopic hook scissors, which were introduced through T2, the needle was removed through T3 and the telescope was used to evaluate the gastropexy.

Then, the same laparoscopic technique was used to perform the ovariectomy in both groups (Bendinelli, Properzi, et al., 2019). Each ovary was grasped at the level of the proper ligament using Wolf grasping forceps (Wolf Medical Instrument), and the ovarian pedicles were sealed and cut using a bipolar vessel sealing device (PL720SU‐Caiman, Aesculap, B. Braun). Both ovaries were removed through T2. Then, carbon dioxide was evacuated from the abdominal cavity by gently compressing the abdomen, and the portals were removed. The abdominal fascia of the umbilical port, external abdominal oblique muscle and subcutaneous tissues were closed in separate layers with 2‐0 glyconate (Monosyn, B. Braun) in a simple continuous pattern. The skin was closed with a tissue adhesive (Vet Bros Company). Surgical time, defined as the time from the first skin incision to the time of the skin closure with the tissue adhesive, was recorded.

Major and minor intraoperative and postoperative complications were recorded. Major intraoperative complications were defined as complications requiring considerable deviation from standard surgical procedure (e.g. emergency conversion to an open surgery because of failure of the suture of the gastropexy, serious damage to viscera or uncontrolled haemorrhage). Minor intraoperative complications were defined as complications that resolved with no considerable deviation from the normal surgical procedure (e.g. minor haemorrhage or suture line break). Major postoperative complications were those that required veterinary intervention during the postoperative period (e.g. surgical site infection or persistent seroma). Minor postoperative complications were defined as self‐limiting complications (e.g. bruising, swelling, erythema or subcutaneous emphysema near the incision sites) (Follette et al., 2019; Spah et al., 2013).

### 2.4 Postoperative pain assessment and management

The Glasgow composite pain scale (short form) was used to assess the degree of pain (Reid et al., 2007). The Glasgow pain score (GPS) was assigned by answering a questionnaire relating to the animal's response to wound palpation and to its behaviour using all variables except mobility because the dogs were taken only for short walks to defecate or urinate every 12 hr. The GPS was calculated by summing the scores from the answers (range, 0−20). The first GPS value was obtained 1 hr before surgery. Afterward, the GPS was calculated at 1, 6, 12, 18 and 24 hr after extubation. The GPS was always performed by the same veterinarian. Patients with GPS ≥ 5 (Reid et al., 2007) were given tramadol (Altadol, Formevet) 3 mg/kg IV as rescue analgesia (Bendinelli, Properzi, et al., 2019).

Food and water were offered 8 hr postoperatively (Mathon et al., 2009). Patients were discharged the day after surgery with cephadroxil (Cefa‐cure tabs, MSD Animal Health) 25 mg/kg orally every 12 hr and meloxicam (Boehringer Ingelheim) 0.1 mg/kg orally once daily for 5 days. Owners were contacted daily by telephone for 10 days and asked about evidence of major and minor complications (Gandini & Giusto, 2016). Each dog had an ultrasound examination of the gastropexy site approximately 6 months postoperatively.

### 2.5 Statistical analysis

Kolmogorov–Smirnov test was performed to verify the normal distribution of the data. The data were normally distributed. Analysis of variance (ANOVA) was performed to evaluate the data using the general linear model procedure of SAS software Version 9.4 with gastropexy technique (two levels: LAG and TLG) as a fixed factor. The proportion of animals in each group that required rescue analgesia and that were diagnosed with complications was submitted to the chi‐square test with Yates correction due to the low number of observations. The Glasgow pain scores were reported as least‐squares means corrected for age ± *SE*. Age, weight and surgical time were expressed as the mean ± standard deviation (*SD*). Statistical significance was set at *p* < .05.

## 3 RESULTS

### 3.1 Animals

Twenty‐eight dogs were enrolled in the study. Of these dogs, 14 were assigned to the LAG group and 14 to the TLG group. Two dogs of the LAG group were withdrawn from the study because the uterus was hyperplastic, and the laparoscopic procedure was converted into open surgery to perform ovariohysterectomy and gastropexy. The remaining 12 dogs of the LAG group included mixed breed dogs (*n* = 5), Labrador retrievers (*n* = 3), golden retrievers (*n* = 2), cane corso (*n* = 1) and Central Asian shepherd dog (*n* = 1). The 14 dogs of the TLG group included mixed breed dogs (*n* = 2), Saint Bernard (*n* = 2), golden retrievers (*n* = 2), great Dane (*n* = 1), French mastiff (*n* = 1), grey ghost Weimaraner (*n* = 1), Australian shepherd dog (*n* = 1), Labrador retriever (*n* = 1), Rhodesian ridgeback (*n* = 1), Staffordshire bull terrier (*n* = 1) and German boxer (*n* = 1). No significant differences regarding age and weight were recorded between groups (Table 1).

**Table 1:** Demographic data (age and body weight) and surgical time in both groups

	LAG group	TLG group	*p* value
Age, months (mean ± *SD*)	19.4 ± 3.9	14.2 ± 3.6	.33
Body weight, kg (mean ± *SD*)	27.5 ± 3.6	32.7 ± 3.3	.30
Surgical time, min (mean ± *SD*)	39 ± 2^a^	48 ± 2^b^	.004

Note

Values are mean ± standard deviation. Superscript letters (a, b) in the same row indicate significant difference between groups.

Abbreviations: LAG, laparoscopic‐assisted gastropexy; TLG, total laparoscopic gastropexy.

### 3.2 Surgical time, complications and follow‐up

Laparoscopic procedures were completed successfully in all dogs (*n* = 26). Surgical time was significantly longer in the TLG group compared with the LAG group (Table 1). No major or minor intraoperative complications were encountered. Minor postoperative complications were documented in two dogs belonging to the LAG group and in one dog belonging to the TLG group with no significant difference regarding the incidence of complications between groups (chi‐square: 0.02, *p* = .88). In the LAG group, swelling near the site of gastropexy was diagnosed in one dog at 24 hr immediately before discharge and in one dog at 7 d by phone interview. The dog belonging to the TLG group was diagnosed with subcutaneous emphysema near the site of insertion of T1 at 24 hr immediately before discharge. The owners were contacted daily by phone and asked about the evolution of the complications. No dogs required an examination. Swelling and subcutaneous emphysema spontaneously resolved within 3 d after onset.

In all dogs, ultrasound examination at 6 months postoperatively highlighted that the right abdominal wall was in contact with the right side of the stomach, and no sliding motion between the stomach and the body wall was detected at the gastropexy site. The clips were visible at the gastropexy site and were surrounded by a thin and avascular capsule.

### 3.3 Postoperative pain

There were no significant differences regarding the GPS values between groups (Table 2). In both groups, the GPS significantly increased at 1 hr (*P*_LAG_ = 0.0001; *P*_TLG_ = 0.001) and 6 hr (*P*_LAG_ = 0.0001; *P*_TLG_ = 0.0001) compared with GPS before surgery (Table 2). In both groups, the GPS significantly decreased at 12 hr (*P*_LAG1h_ = 0.0067; *P*_LAG6h_ = 0.0017; *P*_TLG_ = 0.002), 18 hr (*P*_LAG1h_ = 0.0006; *P*_LAG6h_ = 0.0001; *P*_TLG1h_ = 0.0033; *P*_TLG6h_ = 0.0002) and 24 hr (*P*_LAG_ = 0.0001; *P*_TLG_ = 0.001) compared with GPS at 1 hr and 6 hr. Two dogs in each group required rescue analgesia with no significant difference regarding the number of dogs rescued between groups (chi square: 0.028, *p* = .87). In the LAG group, one dog was rescued at 1 hr and one at 18 hr. In the TLG group, two dogs were rescued at 6 hr.

**Table 2:** Glasgow pain score (least‐squares means ± standard error) in both groups

Time	LAG group	TLG group	*p* value (in the row)
Before surgery	0.50 ± 0.19^a^	0.57 ± 0.17^a^	.78
1 hr	2.67 ± 0.39^b^	1.99 ± 0.36^b^	.22
6 hr	2.85 ± 0.43^b^	2.35 ± 0.41^b^	.39
12 hr	1.50 ± 0.35^a^	1.49 ± 0.33^a^	.97
18 hr	0.98 ± 0.34^a^	0.65 ± 0.32^a^	.48
24 hr	0.33 ± 0.16^a^	0.21 ± 0.15^a^	.58

Note

Superscript letters (a, b) in the same column indicate significant difference (*p* < .05) among times.

Abbreviations: LAG, laparoscopic‐assisted gastropexy; TLG, total laparoscopic gastropexy.

To avoid the influence of tramadol on the GPS, the data regarding dogs receiving rescue analgesia (*n* = 4) were withdrawn (Table 3). Even after the withdrawal of rescued dogs, there were no significant differences in GPS between groups.

**Table 3:** Glasgow pain score (least‐squares means ± standard error) excluding dogs that received tramadol

Time	LAG group	TLG group	*p* value (in the row)
Before surgery	0.50 ± 0.19^a^ (12)	0.57 ± 0.17^a^ (14)	0.78
1 hr	2.67 ± 0.39^b^ (12)	1.99 ± 0.36^b^ (14)	0.22
6 hr	2.92 ± 0.46^b^ (11)	2.35 ± 0.41^b^ (14)	0.37
12 hr	1.36 ± 0.33^a^ (11)	1.34 ± 0.32^a^ (12)	0.96
18 hr	1.01 ± 0.38^a^ (11)	0.66 ± 0.36^a^ (12)	0.51
24 hr	0.29 ± 0.17^a^ (10)	0.17 ± 0.16^a^ (12)	0.58

Note

In parentheses, the number of dogs that did not receive tramadol. Superscript letters (a, b) in the same column indicate significant difference (*p* <.05) among times.

Abbreviations: LAG, laparoscopic‐assisted gastropexy; TLG, total laparoscopic gastropexy.

## 4 DISCUSSION

Laparoscopic OIE may be safely combined with LAG or TLG. Combined laparoscopic OIE and TLG took longer to perform than combined laparoscopic OIE and LAG. No differences regarding postoperative pain score within the first 24 hr were observed between groups, and the incidence of complications in both groups was comparable, even though both techniques did differ substantially.

Although a three‐port technique is not commonly needed to perform a LAG and therefore results from this study may not be consistent among other techniques for performing LAG, we recorded lower surgical times than those previously reported in dogs undergoing combined laparoscopic OIE and gastropexy (Gandini & Giusto, 2016; Rivier et al., 2011; Runge & Mayhew, 2013). Furthermore, in this study, the surgical time of combined laparoscopic OIE and LAG was approximately 20% shorter than the surgical time of combined laparoscopic OIE and TLG. Reduced surgical times may be related to inclusion criteria. In fact, we enrolled only dogs with a BCS 3/5 because in obese patients undergoing abdominal procedures, surgical access is often difficult, and surgical and anaesthetic complications increase (Sloth, 1992). Additional studies will be needed to determine whether initial BCS is related to surgical time. Although the surgical time of gastropexy itself was not recorded, the difference between groups regarding surgical time may be related to the surgeon's experience and to the suturing technique. Although the surgeon had significant clinical practice with both techniques, the extra‐abdominal suturing technique for LAG is undoubtedly more simple and rapid to perform than the intracorporeal suturing technique for TLG (Coleman & Monnet, 2017; Mayhew & Cimino, 2009; Spah et al., 2013; Takacs et al., 2017). In the TLG group, it is also likely that the barbed suture enabling further reduction in surgical time (Spah et al., 2013). Moreover, the use of an additional titanium biocompatible endoclip to reinforce the suture is safe and not time consuming (Guedes et al., 2017; Palmisano et al., 2014).

Although OIE is most commonly performed first (Gandini & Giusto, 2016; Rivier et al., 2011), we performed gastropexy first, and no technical difficulties were detected. The laparoscopic procedure was converted into open celiotomy to perform ovariohysterectomy in two dogs because the uterus was hyperplastic. To avoid this complication, the authors recommend performing ultrasound examination of the uterus before minimally invasive ovariectomy.

No other previously reported intraoperative complications (e.g. splenic laceration, haemorrhage) were encountered in either group (Mayhew & Cimino, 2009; Runge & Mayhew, 2013; Takacs et al., 2017). These complications may occur during initial port entry because of loss of triangulation and restricted range of instrument motion internally (Runge & Mayhew, 2013). According to the authors’ opinion, the use of a multi‐port technique may help the surgeon avoid these complications and other technical difficulties (Mayhew & Cimino, 2009).

Only self‐limiting postoperative complications were recorded. As expected, swelling near the site of gastropexy was the most common postoperative complication recorded in this study (Bendinelli, Properzi, et al., 2019; Loy Son et al., 2016; Rivier et al., 2011). It is likely that swelling recorded in dogs belonging to the LAG group was caused by the body's natural response to skin incision and subcutis dissection, although we cannot exclude that it was also related to extracorporeal sutures of polyglactin, which usually causes more tissue reaction compared with polydioxanone (Mathon et al., 2009; Rasmussen, 2003). Subcutaneous emphysema, an uncommon complication after laparoscopic surgery, was detected in a dog belonging to the TLG group, and it was probably related to dissection of the insufflated CO_2_ from the peritoneal cavity to the subcutaneous tissue at the trocar site (Rasmussen, 2003).

The incidence of self‐limiting postoperative complications in both groups was lower compared with that previously reported (Loy Son et al., 2016; Rivier et al., 2011) and unlike that previously reported (Mayhew & Cimino, 2009), comparable with both techniques. We hypothesized that laparoscopic surgical procedures performed by a specialist result in less inflammation of the incisional site than those performed by an inexperienced surgeon (Bendinelli, Properzi, et al., 2019; Haraguchi et al., 2017; Loy Son et al., 2016; Mathews, 2000), even though, in the veterinary literature, there are no studies that evaluate the amount of inflammation of the incisional site related to the surgeon's experience with laparoscopy.

Postoperative pain mainly occurs over the first 24 hr after tissue injury (Bendinelli, Properzi, et al., 2019; Hancock et al., 2005). Patients undergoing laparoscopic procedures may experience pain related to the number of ports (Case, Marvel, Boscan, & Monnet, 2011), surgical procedure and technique (Bendinelli, Properzi, et al., 2019; Haraguchi et al., 2017), surgical time (Bendinelli, Properzi, et al., 2019) and carbon dioxide insufflation of the abdomen (Woehlck et al., 2003). Laparoscopic gastropexy and OIE may be performed using a single‐port or multi‐port technique (Bendinelli, Properzi, et al., 2019; Gandini & Giusto, 2016; Mayhew & Cimino, 2009; Rasmussen, 2003; Rivier et al., 2011; Runge & Mayhew, 2013; Spah et al., 2013). The single‐port technique decreases the number of incisions and surgical trauma compared with the multi‐port technique (Runge & Mayhew, 2013). Nevertheless, the single‐port technique has an inherent disadvantage because of the loss of triangulation, which may increase surgical time and complication rates (Gandini & Giusto, 2016). We preferred a three‐port technique because it ensures the independence of the laparoscope from the instruments and because it has been demonstrated that the total pain score for dogs with a single‐port procedure did not differ significantly from scores for dogs with multiple ports (Case et al., 2011). In addition, the laparoscopic gastropexy technique (e.g. full‐thickness incision of the body wall) plays an important role in postoperative pain, as LAG is undoubtedly more invasive than TLG (Mathon et al., 2009; Mayhew & Cimino, 2009; Takacs et al., 2017). However, we performed LAG through skin incision and subcutis dissection (Mathon et al., 2009) because it is less invasive than LAG performed through a full‐thickness incision of the body wall (Haraguchi et al., 2017). Nevertheless, the technique we used must be performed very carefully because there is the risk of penetrating the gastric lumen. Regarding the influence of surgical time on postoperative pain, extended surgical times often increase the severity of postoperative pain after laparoscopic surgery (Case et al., 2011). In this study, although LAG was performed faster than TLG, there were no significant differences regarding the degree of postoperative pain between groups. Finally, postoperative pain may be related to insufflation of the abdomen with carbon dioxide, which causes mechanical pain because of abdominal distension and the formation of carbonic acids that act on peritoneal surfaces. It is always attempted to remove carbon dioxide as much as possible but it is impossible to remove completely. Therefore, it is not likely that the careful evacuation was what prevented these side effects from occurring (Woehlck et al., 2003).

In light of the above findings, evaluation and treatment of postoperative pain are critical in dogs undergoing combined laparoscopic OIE and laparoscopic gastropexy. Previous studies underscored that LAG resulted in low postoperative pain assessed with the Melbourne Pain Scale and visual analogue scale (Haraguchi et al., 2017; Mathon et al., 2009), and that TLG was associated with improvement in the willingness of dogs to move around postoperatively compared with LAG (Mayhew & Cimino, 2009). Unfortunately, to the best of our knowledge, the relationship between dog activity and postoperative pain has not been clearly demonstrated to date (Culp, Mayhew, & Cimino, 2009). In this study, by performing frequent pain assessments using a standard pain scoring system by a single investigator, we wanted to demonstrate that dogs undergoing combined laparoscopic OIE and TLG experienced less postoperative pain compared with dogs undergoing combined laparoscopic OIE and LAG. Contrary to what was expected, no difference in GPS was recorded between groups. We hypothesized that extended surgical time of the TLG group may have counterbalanced the greater invasiveness of LAG. It is likely that extended surgical time of the TLG group allowed carbon dioxide to produce carbonic acids that acted on peritoneal surfaces (Woehlck et al., 2003) for longer compared with the LAG group. However, as expected, the GPS values highlighted that both procedures were associated with mild postoperative pain, even though approximately 15% of dogs required rescue analgesia. According to our results and similar to previous findings (Coleman & Monnet, 2017; Loy Son et al., 2016; Runge & Mayhew, 2013), it is advisable that dogs undergoing combined laparoscopic OIE and LAG or TLG receive at least one additional bolus of an opioid postoperatively to reduce the peak of pain between 1 and 6 hr postoperatively.

There are several limitations of this study. First, the major limitation is the non‐blinded nature of the pain assessment. Dressing could have been applied to the abdomen to perform a blind study. Nevertheless, bandages would have made the evaluation of the surgical sites difficult and could have been stressful to the dogs, distorting the data. However, postoperative pain evaluation is always critical. Postoperative pain in dogs undergoing prophylactic gastropexy has been previously measured using the visual analogue scale, University of Melbourne Pain Scale and GPS (Bendinelli, Properzi, et al., 2019; Haraguchi et al., 2017). All these methods are subjective assessments of postoperative pain and are susceptible to errors of under‐ or overestimation (Reid et al., 2007). The major limitation of the Glasgow Composite Pain Scale (short form) is the “mobility” category because its assessment could not always be carried out (Reid et al., 2007). Therefore, it will be necessary to evaluate postoperative pain using a combination of these subjective scales and measurement of objective parameters (e.g. serum cortisol, plasma C‐reactive protein and mechanical nociceptive threshold tested by using von Frey filaments) (Case et al., 2011; Haraguchi et al., 2017). Second, the two techniques are notably different. It would be desirable to make laparoscopic surgical techniques as comparable as possible using the same suture material to perform gastropexy. Afterwards, further studies will be required to demonstrate irrefutably that the incidence of postoperative complications is comparable for these techniques and to correlate the type of complication with the laparoscopic gastropexy technique. Third, even though the ultrasonographic appearance of gastropexy sites at 6 months postoperatively is comparable with previous findings (Rivier et al., 2011), longer‐term follow‐up to assess the efficacy of prophylactic gastropexy by ultrasound examination would be necessary.

## 5 CONCLUSIONS

Combined laparoscopic OIE and TLG is associated with extended surgical times compared with combined laparoscopic OIE and LAG. A moderate incidence of self‐limiting postoperative complications may occur with both techniques. Although combined laparoscopic OIE and LAG or TLG is a minimally invasive procedure and is associated with reduced postoperative pain, it is advisable for dogs undergoing combined laparoscopic OIE and LAG or TLG to receive at least one additional bolus of an opioid after the surgery.

## CONFLICT OF INTEREST

The authors declare that they have no conflicts of interest.

## AUTHOR CONTRIBUTIONS

FL, CB, and PB designed the study. RP, CB and PB performed anaesthetic and surgical procedures. CB performed the postoperative assessment. FL, JR, SP and GLC analysed and interpreted the data. FL, JR, SP and GLC drafted the manuscript. All authors read and approved the final manuscript.

## ETHICAL STATEMENT

The authors confirm that the ethical policies of the journal, as noted on the journal's author guidelines page, have been adhered to and the appropriate ethical review committee approval has been received.
